# Deep learning reveals determinants of transcriptional infidelity at nucleotide resolution in the allopolyploid line by goldfish and common carp hybrids

**DOI:** 10.1093/bib/bbaf260

**Published:** 2025-06-05

**Authors:** Kaizhuang Jing, Tingchu Wei, Xuedie Gu, Guoliang Lin, Lin Liu, Jing Luo

**Affiliations:** School of Information, Yunnan Normal University, Kunming 650500, China; State Key Laboratory for Conservation and Utilization of Bio-resource, School of Ecology and Environment, School of Life Sciences, Yunnan University, Kunming 650091, China; Southwest United Graduate School, Kunming 650092, China; State Key Laboratory for Conservation and Utilization of Bio-resource, School of Ecology and Environment, School of Life Sciences, Yunnan University, Kunming 650091, China; State Key Laboratory for Conservation and Utilization of Bio-resource, School of Ecology and Environment, School of Life Sciences, Yunnan University, Kunming 650091, China; School of Information, Yunnan Normal University, Kunming 650500, China; State Key Laboratory for Conservation and Utilization of Bio-resource, School of Ecology and Environment, School of Life Sciences, Yunnan University, Kunming 650091, China; Southwest United Graduate School, Kunming 650092, China

**Keywords:** transcriptional infidelity, the allopolyploid line by goldfish and common carp hybrids, transcription factors

## Abstract

During DNA transcription, the central dogma states that DNA generates corresponding RNA sequences based on the principle of complementary base pairing. However, in the allopolyploid line by goldfish and common carp hybrids, there is a significant level of transcriptional infidelity. To explore deeper into the causes of transcriptional infidelity in this line, we developed a deep learning model to explore its underlying determinants. First, our model can accurately identify transcriptional infidelity sequences at the nucleotide resolution and effectively distinguish transcriptional infidelity regions at the subregional level. Subsequently, we utilized this model to quantitatively assess the importance of position-specific motifs. Furthermore, by integrating the relationship between transcription factors and their recognition motifs, we unveiled the distribution of position-specific transcription factor families and classes that influence transcriptional infidelity in this line. In summary, our study provides new insights into the deeper determinants of transcriptional infidelity in this line.

## Introduction

In 1956, Francis Crick proposed the central dogma [1], which illustrates the pathway of genetic information transfer among biological macromolecules. According to the central dogma, DNA serves not only as a template for its own replication but also as a template for RNA synthesis, which is the core process of transcription. Subsequently, RNA acts as a template guiding protein synthesis, forming the basis of the translation process [1, 2]. The central dogma indicates that the transfer of genetic information, whether between different generations or between different cells within the same organism, generally maintains a high level of fidelity, ensuring the stability of genetic information across species. However, during actual transcription, the DNA sequence should generate corresponding RNA sequences based on the principle of complementary base pairing, but transcriptional infidelity may still occur. This phenomenon refers to the generation of RNA sequences that do not completely match the template DNA sequence during transcription (i.e. RNA-DNA sequence differences), thereby violating the principle of complementary base pairing [3–5]. Previous studies on transcriptional infidelity in eukaryotic organisms (such as *Saccharomyces cerevisiae* and human B cells) have found that the transcriptional error rate can reach up to the order of ${10}^{-4}$ [6–8].

Transcriptional infidelity becomes increasingly pronounced in the allopolyploid line by goldfish and common carp hybrids. This line represents a vertebrate distant hybrid polyploidization model, where the maternal parent is the goldfish (*Carassius auratus* red var., $2n = 4x = 100$), and the paternal parent is the common carp (*Cyprinus carpio* L., $2n = 4x = 100$). The first two generations of hybrids are tetraploid ($2n = 4x = 100$), while subsequent generations are octoploid ($2n = 8x = 200$) [9, 10]. In previous studies, Professor Jing Luo's team from Yunnan University conducted resequencing and transcriptome analyses on progeny with different ploidy levels of the allopolyploid line, covering adult tissues from 12 individuals and 8 tissue types. The study measured transcriptional infidelity levels and found that transcriptional infidelity in this line reached a magnitude of ${10}^{-3}$, which is significantly higher than the general level. Elevated transcriptional infidelity could lead to reduced proteostasis and affect various biological processes, including nucleotide synthesis, nitrogen metabolism, and tryptophan degradation [8]. Polyploidization occurs in only a small number of vertebrate species. The stable allopolyploid line used in this study provides a unique opportunity to investigate the causes of this rarity among extant animals. Analysis of the abnormal transcriptional infidelity observed in this line not only yields valuable insights into the molecular mechanisms underlying the scarcity, low survival, and limited reproduction of polyploidized vertebrates, but also establishes a critical theoretical foundation for future research on vertebrate polyploidization.

The mechanisms underlying transcriptional infidelity are highly complex and likely influenced by multiple factors, including the classical RNA editing mechanisms [11] and DNA/RNA damage processes. Moreover, the allopolyploid line may experience ``genome shock'' induced by polyploidization, which itself can trigger frequent transcriptional infidelity events [12]. However, with the advancement of sequencing technologies, it has been discovered that many sites of transcriptional infidelity cannot be explained by classical RNA editing or similar mechanisms. This phenomenon may partly result from technical errors, but it may also reflect genuine biological phenomena [13–15]. Consequently, further experimental evidence is needed in this field to validate and support these findings, leading to a deeper understanding of the mechanisms driving transcriptional infidelity and its impact on biological systems. Previous studies [12] have utilized high-throughput sequencing technologies to uncover several patterns of transcriptional infidelity in this line. These include the characteristics of nucleotide substitutions—among which the four most prevalent types are A-to-G, T-to-C, G-to-A, and C-to-T—the genomic distribution of transcriptional infidelity events, the association between gene expression levels and transcriptional infidelity, and the functional enrichment of genes exhibiting both high expression and high transcriptional infidelity.

However, previous studies at nucleotide resolution have primarily relied on statistical approaches, which are limited in their ability to uncover position-specific pattern features of transcriptional infidelity. To investigate the causes of the abnormally high levels of transcriptional infidelity in this line, we aim to develop a computational method to identify regions of transcriptional infidelity at nucleotide resolution using contextual sequence information from the DNA. We also seek to study local genomic parameters that influence the occurrence of transcriptional infidelity, thereby providing molecular insights into the mechanisms behind transcriptional infidelity on a genome-wide scale. Deep learning methods, particularly genomic pre-trained models, have proven effective in capturing complex relationships and dependencies within DNA sequences [16, 17]. These methods have demonstrated superior performance in various genomics tasks, including polyadenylation site prediction [18] and single-strand break site prediction [19].

Therefore, we have developed the first deep learning model to study transcriptional infidelity at nucleotide resolution. This model can accurately identify DNA sequences within the genome of the allopolyploid line by goldfish and common carp hybrids that exhibit transcriptional infidelity and offers subregional predictions for these infidelity-prone DNA sequences. Additionally, we quantitatively analyze the contribution of motifs within the context of transcriptional infidelity sites and explore the relationship between transcription factors, their families, and classes with the binding motifs, revealing associations between transcriptional infidelity sites and position-specific transcription factor families and classes. In summary, our research offers new perspectives on understanding the mechanisms of transcriptional infidelity in the allopolyploid line by goldfish and common carp hybrids. All abbreviations are defined in the Nomenclature section of the Supplementary File.

## Material and methods

### Source of transcriptional infidelity site data

All transcriptional infidelity site data, along with the reference genome and annotation files, were provided by Professor Jing Luo's team at Yunnan University. The dataset we used had already undergone quality control and genome alignment processing, with classical RNA editing sites removed. We selected kidney cells from F$_{1}$-generation (Filial generation 1) individuals of the allopolyploid line by goldfish and common carp hybrids as the dataset for model training and analysis. In this sample, 3,610,001 transcriptional infidelity sites were detected, among which 118,702 sites exhibited multiple types of infidelity.

### Developing the TTIRI model

#### Input dataset

To enable the model to effectively learn information related to transcriptional infidelity from the DNA sequence context, we constructed the candidate positive samples by sliding a window of 200 nt with a step size of 10 nt across the reference genome, excluding sequences that did not contain transcriptional infidelity sites. For constructing the candidate negative samples, we applied the same sliding method within coding regions, excluding sequences with transcriptional infidelity sites. The candidate positive samples did not use coding regions as a sliding template to avoid a potential loss in the distribution of infidelity site samples due to coding regions being shorter than the specified sample interval length. Finally, we selected sequences containing infidelity sites from the candidate positive samples as positive samples and sequences without infidelity sites from the candidate negative samples as negative samples.

Through these steps, we obtained 20,647,506 positive samples and 2,650,319 negative samples. To avoid introducing unnecessary complexity from a small number of samples containing non-determined bases and to reduce the storage and computational burden during model training, we first filtered out all samples containing the ``N'' character, retaining only sequences composed of the four determined bases ``ATGC''. Considering the significant difference in data volume between positive and negative samples, which could bias the model during training and affect its generalization ability, we randomly selected 10,000 samples from each group to create a balanced dataset. We then split this dataset into training, validation, and test sets in an 8:1:1 ratio to ensure the reliability and stability of model evaluation.

#### Model architecture

The tokenization and embedding learning foundation of the TTIRI model adopts the approach of the DNABERT-2 model [20], utilizing the pre-training and fine-tuning paradigms provided by this model. The concept of DNABERT-2 is inspired by the BERT model [21] from the field of natural language processing (NLP), which is a contextual language representation model based on the Transformer architecture [22]. The model is initially pre-trained on a large amount of unlabeled data to develop general understanding and then fine-tuned under the guidance of specific tasks to address various task-specific data applications. The effectiveness of DNABERT-2 has been validated across multiple bioinformatics problems, with its performance comparable to the current state-of-the-art models [17, 23–25].

**Figure 1:**
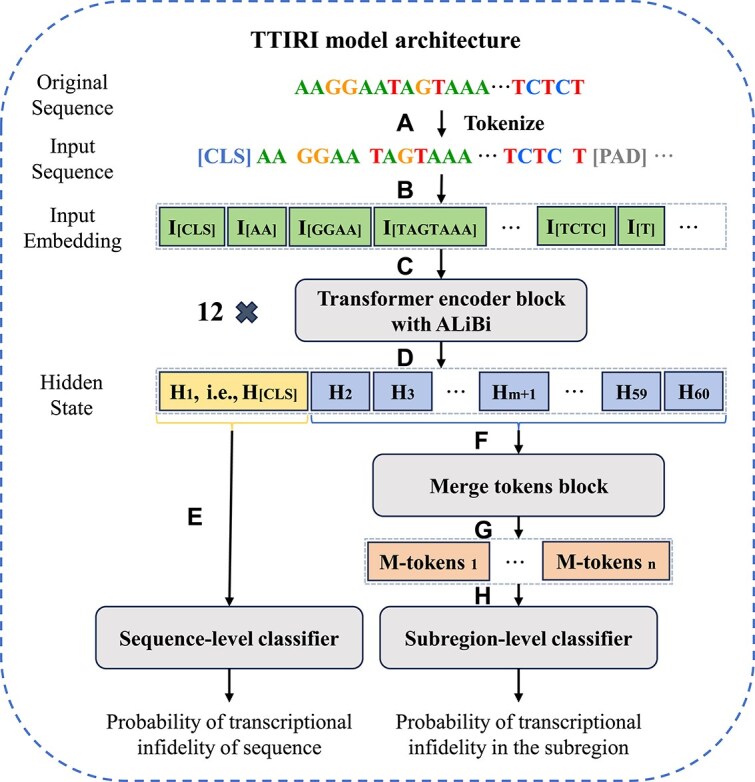
TTIRI model architecture. (A) Tokenization of the original sequence into tokens. (B) Conversion of tokens into numerical vectors. (C) The numerical matrix is fed into the Transformer encoder block with ALiBi for contextual learning. (D) Acquisition of new hidden representations enriched with contextual information. (E) The CLS token is passed to the sequence-level classifier. (F and G) The remaining tokens are passed to the merging token block to obtain m-tokens representing subregions. (H) The m-tokens are passed to the subregion-level classifier.

For an input sequence of 200 nt, the model first tokenizes the DNA sequence using a combination of SentencePiece [26] and Byte-Pair Encoding (BPE) [27] (Fig. 1A). SentencePiece is a language-agnostic tokenizer that treats each input as a raw stream without assuming any pre-tokenization. BPE is a compression algorithm that learns a fixed-size, variable-length token vocabulary based on the co-occurrence frequency of characters and is widely used as a tokenization strategy in NLP. The specific process of BPE tokenization begins with initializing the vocabulary with all unique characters in the corpus. Then, in each iteration, the most frequently occurring a pair of characters is identified and added to the vocabulary as a new token, replacing all identical character pairs in the corpus with this new token. This process is repeated until the desired vocabulary size is constructed. Through this method, BPE effectively captures patterns and structures within DNA sequences, providing richer representations for downstream tasks. The vocabulary was empirically derived using the DNABERT-2 model, with a vocabulary size of 4096. Given the variable length of the tokens in the vocabulary, a 200 nt sequence, after tokenization, is approximately reduced to one-fifth of its original length, or about 40 effective tokens, as evaluated by the median token count across all samples in the dataset. After obtaining the effective tokens, special tokens such as CLS, SEP, and padding tokens are added to facilitate subsequent sequence classification and to ensure consistency in the total number of tokens per sequence. We denote the total number of tokens as ${{n}_{t}}$ (${{n}_{t}}=60$).

Following tokenization, samples are transformed into token representations. These tokens are then passed to the embedding layer (Fig. 1B), which converts each token in the sequence into a corresponding numerical vector, representing each sequence as a matrix $M\in{{\mathbb{R}}^{{{n}_{t}}\times{{d}_{h}}}}$, where ${{d}_{h}}$ (${{d}_{h}}=768$) denotes the size of the hidden layer, i.e. the dimension of the embedded numerical vectors. Next, matrix $M$ is passed to a stack of 12 Transformer encoder blocks (Fig. 1C), each containing 768 hidden units and 12 attention heads. Positional embeddings are incorporated through the attention with linear biases (ALiBi) mechanism [28]. Formally, each encoder block captures the contextual information of $M$ through the multi-head self-attention mechanism:


\begin{gather*} MultiHead(M)=\operatorname{Concat}(Hea{{d}_{1}},...,Hea{{d}_{h}}){{W}^{O}} \nonumber \\ Hea{{d}_{i}}=\operatorname{Attention}({{Q}_{i}},{{K}_{i}},{{V}_{i}})=\operatorname{softmax}\left(\frac{{{Q}_{i}}K_{i}^{T}}{\sqrt{{{d}_{k}}}}+{{B}_{i}}\right){{V}_{i}} \nonumber \\{{Q}_{i}}=MW_{i}^{Q};{{K}_{i}}=MW_{i}^{K};{{V}_{i}}=MW_{i}^{V} \nonumber \end{gather*}

where ${{W}^{O}}\in{{\mathbb{R}}^{{{d}_{h}}\times{{d}_{h}}}}$ and $\{W_{i}^{Q},W_{i}^{K},W_{i}^{V}\}_{i=1}^{h}\in{{\mathbb{R}}^{{{d}_{h}}\times{{d}_{k}}}}$ are learnable weight matrices, $h$ ($h=12$) represents the number of self-attention heads, ${{d}_{k}}$ (${{d}_{k}}=64$) is the column vector dimension of ${{Q}_{i}}$ and ${{K}_{i}}$, and ${{B}_{i}}\in{{\mathbb{R}}^{{{n}_{t}}\times{{n}_{t}}}}$ denotes the linear bias matrix calculated using the ALiBi method. When computing the next hidden state of $M$, each attention head first calculates the attention scores between every pair of tokens, then uses these scores as weights to perform a weighted sum over the rows of ${{V}_{i}}$, thereby generating a new hidden representation for each token in the sequence that reflects the contextual tokens (Fig. 1D).

After learning through the 12 stacked encoder blocks, the model passes the CLS token of each sequence to the sequence-level classifier (Fig. 1E) to predict transcriptional infidelity phenomena at the sequence-level. Simultaneously, the model merges the remaining tokens of each sequence (denoted as ${{M}^{t}}\in{{\mathbb{R}}^{({{n}_{t}}-1)\times{{d}_{h}}}}$ for any given sequence) and passes them to the subregion-level classifier (Fig. 1F–H) to predict transcriptional infidelity at the subregion-level. In the sequence-level classifier, we optimize using the binary cross-entropy loss function:


\begin{gather*} {{\ell} _{_{sequence}}}=-\frac{1}{N}\sum\limits_{i=1}^{N}{({{y}_{i}}\log ({{p}_{i}})+(1-{{y}_{i}})\log (1-{{p}_{i}}))} \nonumber \end{gather*}

where $N$ denotes the batch size, ${{y}_{i}}\in \{0,1\}$ represents the true label of the sample (0 for normal transcription sequence, 1 for transcriptional infidelity sequence), and ${{p}_{i}}\in [0,1]$ is the predicted probability that the sample is a transcriptional infidelity sequence.

In the subregion-level prediction, adjacent tokens are first merged through non-overlapping max-pooling and average-pooling, then the results of these two methods are concatenated and dimensions are restored to obtain improved hidden representations of subregions (containing one or more tokens). The new representation of any sequence after token merging is denoted as ${{M}^{m}}\in{{\mathbb{R}}^{\widehat{{{n}_{t}}}\times{{d}_{h}}}}$, where $\widehat{{{n}_{t}}}=\left \lceil \frac{{{n}_{t}}}{m} \right \rceil $, $\text{m}\in \text{} \!\!\{\!\!\text{ 1,2,}...\text{,}{{\text{n}}_{t}}\text{-1} \}\!\!\text{ } \!$ denotes the pooling window size (Fig. 1G). The merging process is formalized as follows:


\begin{gather*} {{M}^{m}}=\operatorname{Concat}(\operatorname{Mean}({{M}^{t}},m),\operatorname{Max}({{M}^{t}},m)){{W}^{C}} \nonumber \end{gather*}

where $\{\text{Mean}({{M}^{t}},m),\text{Max}({{M}^{t}},m)\}\in{{\mathbb{R}}^{\widehat{{{n}_{t}}}\times{{d}_{h}}}}$ denote non-overlapping (i.e. stride equal to $m$) average and max pooling over ${{M}^{t}}$ with a window size of $m$, and ${{W}^{C}}\in{{\mathbb{R}}^{2{{d}_{h}}\times{{d}_{h}}}}$ is a learnable weight matrix for restoring the hidden layer dimensions. Each new token in ${{M}^{m}}$ is referred to as an m-token (i.e. a subregion), where m indicates the number of merged tokens. ${{M}^{m}}$ is then passed to the subregion-level classifier (Fig. 1H) to predict whether each subregion exhibits transcriptional infidelity. Due to the class imbalance between positive and negative categories in subregion-level prediction, we optimize using a weighted binary cross-entropy loss function:


(1) \begin{gather*} {{\ell} _{token}} = \frac{-\sum\limits_{i=1}^{N}{\sum\limits_{t=1}^{\widehat{{{n}_{t}}}}{{{V}_{i,t}}\left(w \cdot{{y}_{i,t}} \log{{p}_{i,t}} + (1 - {{y}_{i,t}})\log(1 - {{p}_{i,t}})\right)}}}{\sum\limits_{i=1}^{N}{\sum\limits_{t=1}^{\widehat{{{n}_{t}}}}{{{V}_{i,t}}}}} \nonumber \\ w = \left\{ \begin{aligned} & \frac{\sum\nolimits_{i=1}^{N}{\sum\nolimits_{t=1}^{\widehat{{{n}_{t}}}}{{{V}_{i,t}}({{y}_{i,t}}=0)}}}{\sum\nolimits_{i=1}^{N}{\sum\nolimits_{t=1}^{\widehat{{{n}_{t}}}}{{{V}_{i,t}}({{y}_{i,t}}=1)}}}, \text{if} \sum\limits_{i=1}^{N}{\sum\limits_{t=1}^{\widehat{{{n}_{t}}}}{{{V}_{i,t}}({{y}_{i,t}}=1)}}>0 \nonumber \\ & \sum\limits_{i=1}^{N}{\sum\limits_{t=1}^{\widehat{{{n}_{t}}}}{{{V}_{i,t}}({{y}_{i,t}}=0)}}, \text{otherwise} \nonumber \end{aligned} \right. \end{gather*}

where ${{V}_{i,t}}\in \{0,1\}$ indicates whether a subregion is valid (i.e. the merged original tokens are not all padding tokens), ${{y}_{i,t}}\in \{0,1\}$ represents the true label of the subregion, and ${{p}_{i,t}}\in [0,1]$ denotes the predicted probability of transcriptional infidelity in the subregion.

The final model loss is composed of both sequence and subregion predictions, formally expressed as:


\begin{gather*} \mathcal{L}=\lambda{{\ell} _{_{sequence}}}+(1-\lambda ){{\ell }_{token}} \nonumber \end{gather*}

where $\lambda \in (0,1)$ is a hyperparameter used to balance the two loss components. In our experiments, we chose $\lambda $ to be 0.2 based on the evaluation results of the combined predictions.

#### Model evaluation

In this study, we chose AUROC and AUPRC as evaluation metrics due to their significant effectiveness in assessing classification model performance. These metrics provide an overall measure of performance across all possible classification thresholds, ensuring a comprehensive evaluation of the model's performance.

First, we selected AUROC as the evaluation metric for sequence-level predictions, mainly for the following reasons: AUROC measures the overall ability of the model to distinguish between positive and negative samples, providing a summary of the model's performance across various decision thresholds. For balanced datasets, AUROC accurately reflects the model's overall discriminative ability, with a range from 0 to 1, where 0.5 indicates a random model and 1 indicates a perfect model. A higher AUROC value indicates better performance in distinguishing between positive and negative classes.

Secondly, for subregion-level predictions, we selected AUPRC as the evaluation metric due to the class imbalance in our dataset. AUPRC, which is based on precision and recall, better reflects the model's predictive ability on minority class samples. Precision measures the proportion of true positive samples among all samples predicted as positive, while recall measures the proportion of correctly predicted positive samples among all actual positive samples. AUPRC is particularly useful when the costs of false positives and false negatives differ, as it can identify the optimal classification threshold that minimizes cost. By using AUPRC, we can more comprehensively evaluate the model's performance when handling imbalanced datasets.

### Quantitative analysis of the contribution of position-specific motifs to transcriptional infidelity sites

To interpret the regulatory parameters involved when the model identifies transcriptional infidelity at the genomic level, we first quantified the contribution of position-specific hexamers in Section 2.3.1. However, given the complexity and inefficiency of directly analyzing all 4096 possible hexamers, an effective motif screening or classification method is necessary. Therefore, in Section 2.3.2, we further screened the contributions of position-specific hexamers using transcription factor motifs to extract key motifs closely related to transcriptional infidelity. Finally, in Section 2.3.3, we categorized and analyzed these motifs according to transcription factor families and classes, thereby revealing the regulatory roles of different transcription factor families and classes in transcriptional infidelity.

#### Quantifying the impact of position-specific hexamers on transcriptional infidelity sites

To identify the motifs driving the formation of genome-wide transcriptional infidelity sites and to study the impact of position-specific hexamers on the occurrence of transcriptional infidelity, we conducted the following experiments. We randomly sampled 10,000 transcriptional infidelity sites and obtained the sequences 100nt upstream and downstream of these sites, forming 201 nt-long transcriptional infidelity sequences (with the central position confirmed to have transcriptional infidelity). Next, we constructed 10,000 201nt-long normal transcription sequences using the same method as the TTIRI model input dataset and performed model training and prediction at the sequence-level only. Finally, we analyzed these 10,000 transcriptional infidelity sequences.

We employed motif perturbation to systematically quantify the contribution of position-specific hexamers to the model's prediction of transcriptional infidelity in a sequence. For each hexamer appearing in a transcriptional infidelity sequence, we replaced it 100 times with a random hexamer, keeping the rest of the sequence unchanged. Predictions were made for both the original and perturbed sequences, and the impact of motif perturbation was quantified using the median change in the log-odds of predicted classification probabilities from 100 substitutions. Importance scores for position-specific hexamers in all correctly predicted transcriptional infidelity sequences were calculated as follows:


\begin{gather*} IS_{i,j}^{\text{hexamer}}={{V}_{i}}\cdot \operatorname{median}\left(\Delta \operatorname{logit}_{i,j}^{(1)},...,\Delta \operatorname{logit}_{i,j}^{(C)}\right) \nonumber \\ \Delta \operatorname{logit}_{i,j}^{(k)}=\operatorname{logit}(P({{S}_{i}}))-\operatorname{logit}\left(P\left(S_{i,j}^{(k)}\right)\right) \nonumber \\ \operatorname{logit}(p)=\log \left(\frac{p}{1-p}\right) \nonumber \\ i\in [1,N];j\in [1,L-5];k\in [1,C] \nonumber \end{gather*}

where $N=10,000$ denotes the number of sequences analyzed, $L=201$ represents sequence length, $C=100$ is the number of perturbations, ${{S}_{i}}$ is the $i$ transcriptional infidelity sequence, $S_{i,j}^{(k)}$ is the sequence with the hexamer at the $j$ position of the $i$ sequence randomly perturbed, $P(\cdot )\in (0,1)$ denotes the model's prediction probability, and ${{V}_{i}}\in \{0,1\}$ indicates whether the $i$ sequence was correctly predicted as a transcriptional infidelity sequence (i.e. $P({{S}_{i}})\ge 0.5$, thus ${{V}_{i}}=1$; otherwise, ${{V}_{i}}=0$).

#### Quantifying the impact of position-specific transcription factor binding motifs on transcriptional infidelity sites

To obtain high-quality information on transcription factors and their binding motifs, we selected the latest 2024 version of publicly available, high-quality vertebrate transcription factor data from the Jaspar database [29], which includes 828 transcription factors from 105 families and 44 classes. First, we normalized each transcription factor's position frequency matrix provided by the database using a pseudocount of 0.5 to avoid zero frequencies. Then, we converted the normalized result into a position weight matrix (PWM) using the following formula:


\begin{gather*} PW{{M}_{i,j}}=\log\left (\frac{PP{{M}_{i,j}}}{{{p}_{i}}}\right) \nonumber \\ PP{{M}_{i,j}}=\frac{PF{{M}_{i,j}}+\alpha} {\sum\nolimits_{k}{(PF{{M}_{k,j}}+\alpha )}} \nonumber \end{gather*}

where $i,k\in \{A,C,G,T\}$ represent the four bases, $j\in \{1,2,...,L\}$ represents the position, $L$ is the motif length, and $\alpha =0.5$ is the pseudocount. ${{p}_{i}}$ denotes the background probability, which we assume is equal for the four bases, setting ${{p}_{i}}$ to 0.25.

Next, we used each PWM to align and score each transcriptional infidelity sequence. For a given PWM and sequence to be aligned, we used a sliding window approach with a stride of 1nt to extract subsequences $s$ of the same length as the PWM, and their scores on the PWM were calculated as follows:


\begin{gather*} Score(PWM,s)=\sum\limits_{j=1}^{L}{PW{{M}_{{{s}_{j}},j}}} \nonumber \end{gather*}

where $L$ is the motif length, and ${{s}_{j}}$ denotes the base at the $j$ position of the subsequence. To further standardize these scores, we applied a sigmoid function to map the scores to the range of 0 to 1 and set a threshold of 0.999 to filter the standardized scores to ensure alignment reliability.

Finally, we calculated the impact scores of position-specific transcription factor binding motifs on transcriptional infidelity sites for the correctly identified transcriptional infidelity sequences, formally represented as follows:


\begin{gather*} IS_{i,j,n}^{\text{TF}}=\frac{1}{{{L}_{n}}-5}\sum\limits_{k=0}^{{{L}_{n}}-6}{IS_{i,j+k}^{\text{hexamer}}} \nonumber \\ i\in [1,N];j\in [1,L-5] \nonumber \end{gather*}

where $N$ is the number of sequences, $L$ is the sequence length, $n$ denotes a transcription factor, and ${{L}_{n}}$ is the length of the binding motif for transcription factor $n$.

#### Quantifying the macro impact of transcription factor families and classes in model predictions

To observe the contribution of position-specific motifs to the model's prediction of transcriptional infidelity sequences from a macro perspective, we aggregated the results of the analysis of 10,000 sequences and classified transcription factors according to their families and classes to reduce quantification errors and information redundancy introduced by similar transcription factor binding motifs. The impact scores of position-specific transcription factor families and classes on model predictions were calculated as follows:


\begin{gather*} IS_{j,fn/cn}^{\text{Family/Class}}=\frac{\sum\nolimits_{i=1}^{N}{\sum\nolimits_{n\in fn/cn}{{{V}_{i,j,n}}\cdot IS_{i,j,n}^{\text{TF}}}}}{\sum\nolimits_{i=1}^{N}{\sum\nolimits_{n\in fn/cn}{{{V}_{i,j,n}}}}} \nonumber \\ j\in [1,L-5];{{V}_{i,j,n}}\in \{0,1\} \nonumber \end{gather*}

where $fn/cn$ represents a set of transcription factors of a family or class, and ${{V}_{i,j,n}}$ indicates that the $i$ sequence is a valid prediction and the $j$ position is bound by transcription factor $n$ (in this case, ${{V}_{i,j,n}}=1$, otherwise ${{V}_{i,j,n}}=0$).

## Results

### Development of a deep learning model named TTIRI to identify transcriptional infidelity regions

The original data on transcriptional infidelity sites that we used underwent standard processing, including quality control and genome alignment, and classical RNA editing sites were excluded. To ensure that the model could fully learn the real environment where transcriptional infidelity occurs, we constructed negative samples by sliding and extracting 200 nt of normal transcription sequences in the coding region. Positive samples, on the other hand, were created by sliding and extracting 200 nt sequences containing transcriptional infidelity sites on the reference genome. The choice to use the reference genome as the template for extracting positive samples is due to the finding that about 79% of the coding regions are shorter than the specified 200 nt, but transcriptional infidelity sites within these regions should not be ignored.

Our model constitutes a two-layer transcriptional infidelity region identifier (TTIRI) computational framework (Fig. 1). The backbone component of the model employs the pre-training and fine-tuning structure of the DNABERT2 language model [20], utilizing its excellent embedding learning features to achieve better DNA sequence representation. The model has two output branches: one for predicting the classification probability that a sequence undergoes transcriptional infidelity (sequence-level prediction), and another for predicting the classification probability of transcriptional infidelity occurring in subregions of the sequence (subregion-level prediction). The length of the subregions is not fixed but divided according to the number of tokens. In our default setting, a 200 nt sequence is approximately divided into two subregions (details provided in the methods section). Subregion-level prediction is a further refinement of sequence-level prediction; when the model predicts that a sequence will exhibit transcriptional infidelity, it then proceeds to predict for its subregions. This design allows us to identify regions of transcriptional infidelity at a more detailed nucleotide resolution.

In the sequence-level prediction component, given the balanced nature of the dataset, we use the area under the receiver operating characteristic curve (AUROC) as the evaluation metric. In contrast, for the subregion-level prediction component, where there is significant imbalance in label classes, we employ the area under the precision-recall curve (AUPRC) as the evaluation metric.

### T‌TIRI model accurately predicts transcriptional infidelity regions

We randomly divided the constructed dataset into training, validation, and test sets in an 8:1:1 ratio. During training, only the training set is used to train the model, and all evaluation results are based on the test set. TTIRI is the first computational method designed specifically for predicting transcriptional infidelity sites. Given that its foundation model, DNABERT2, has demonstrated superior performance over baseline machine learning models (such as convolutional neural networks (CNN), long short-term memory networks (LSTM), gated recurrent units (GRU), and their common combinations) across various biological problems, our study focuses on the rationality of the model's architecture and parameter design and their effectiveness in predicting transcriptional infidelity regions. The superiority of the proposed two-layer architecture is validated in the ``Two-layer architecture design advantage'' section of the Supplementary File. The results show that, across varying subregion sizes, the two-layer design achieves an average improvement of $\sim $6 percentage points in subregion-level AUPRC compared to the single-layer design. We next explore the impact of different parameter configurations on model performance and determine the optimal parameter settings.

**Figure 2:**
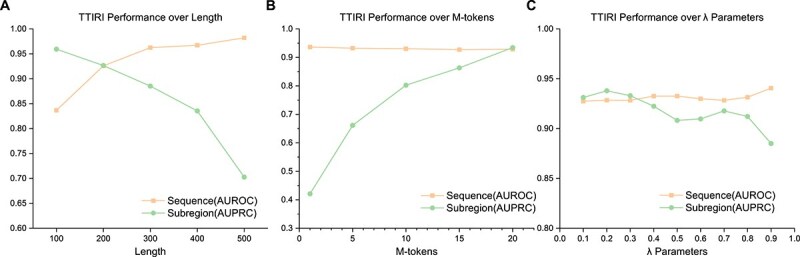
Comparison of TTIRI model performance under different settings. (A) Evaluating the model performance variation with sequence length (from 100 to 500 nt) to determine the optimal sequence length. (B) Evaluating the model performance variation with subregion size (from subregions containing 1 token to 20 tokens) to determine the optimal subregion size. (C) Evaluating the impact of the two-layer classifier loss adjustment hyperparameter $\lambda $ on model performance.

First, we examined the impact of different sequence sample lengths on the model's predictive performance (Fig. 2A). To do this, we created variants of the model's dataset with sequence lengths ranging from 100 nt to 500 nt to identify the optimal sequence length. Figure 2A shows that as sequence length increases, sequence-level prediction performance improves, but subregional-level prediction performance decreases. This phenomenon may be due to the increased sequence length providing richer contextual information, but exacerbating class imbalance within the subregions, thus impairing predictive performance. The experimental results indicate that the model achieves the best overall performance with a sequence length of 200 nt in the two-layer prediction setup.

Next, with the sequence length fixed at 200 nt, we investigated the impact of different subregion sizes on model performance (Fig. 2B). We represented the subregion size by the number of tokens and analyzed cases where the number of tokens ranged from 1 to 20. When the token count was 20, a 200 nt sequence was approximately divided into two subregions. Figure 2B shows that as the number of tokens in a subregion increases, subregional-level prediction performance gradually improves, while sequence-level prediction remains largely unaffected. The model achieves optimal overall predictive performance when the token count is set to 20. However, as the number of tokens increases, although predictive performance improves, the model's ability to identify subregions with finer detail diminishes.

Finally, we studied the effect of the $\lambda $ hyperparameter, which adjusts the relative weight of the losses between the two classifiers during model training, on model performance with a sequence length of 200 nt and a token count of 20 (Fig. 2C). The experimental results show that as the value of $\lambda $ increases, sequence-level prediction performance improves, but subregional-level prediction performance significantly decreases. Considering overall performance, a $\lambda $ setting of 0.2 yields the best results. Under this setting, our model achieved an AUROC of $\sim $0.928 in sequence-level prediction and an AUPRC of $\sim $0.937 in subregional-level prediction. More comprehensive evaluation metrics and detailed numerical results are documented in Supplementary Data 1.

### Quantifying the contribution of position-specific motifs to predicting transcriptional infidelity

Although empirical models can effectively capture the contextual environment in which transcriptional infidelity occurs, the black-box nature of deep learning models makes it challenging to provide biologically insightful analyses of these contexts. Therefore, we employed model interpretability methods to quantify the contribution of position-specific motifs to transcriptional infidelity prediction, aiming to offer biological insights into the occurrence of transcriptional infidelity. Initially, we reconstructed the dataset and conducted sequence-level training and prediction. The positive samples in the new dataset centered on empirically verified transcriptional infidelity sites, while the negative samples represented sequences with normal transcription throughout. This dataset reconstruction was intended to quantify the contribution of position-specific motifs to transcriptional infidelity sites, rather than merely capturing the occurrence region. Subsequently, we evaluated all correctly predicted transcriptional infidelity sequences using a hexamer perturbation approach. For each position in every sequence, we performed 100 perturbations of the hexamer and calculated the median change in the log-odds of the prediction probability before and after the perturbation to determine the contribution score of the position-specific hexamer.

Through the methods described above, we obtained contribution scores for position-specific hexamers within specified sequences. However, due to the large number of hexamer types ($4^{6}=4096$) and the significant influence of noise on individual sequence interpretation scores (including noise introduced by interpretative methods, model fit discrepancies, and inherent dataset noise), we faced challenges in meaningful motif selection. To address this issue, we utilized transcription factors closely associated with the transcription process to identify their binding motifs, thus facilitating more meaningful motif selection. Initially, we downloaded data on vertebrate transcription factors from the 2024 edition of the Jaspar database [29], which includes 828 transcription factors belonging to 105 families and 44 classes. We then scanned all correctly predicted transcriptional infidelity sequences, binding transcription factors to their reliable binding motifs, and combined this with the contribution scores of position-specific hexamers to determine the contribution of position-specific transcription factors (represented by their corresponding binding motifs) to transcriptional infidelity sites. Lastly, to identify the main drivers behind the formation of transcriptional infidelity sites across the transcriptome, we merged the analysis results of all sequences and eliminated the influence of frequency. Detailed scores for transcription factor motif recognition importance and frequency distributions are recorded in Supplementary Data 2. Additionally, we have provided a specific-sequence analysis example in the ``Sequence-Specific Analysis Example'' section of the Supplementary File.

**Figure 3:**
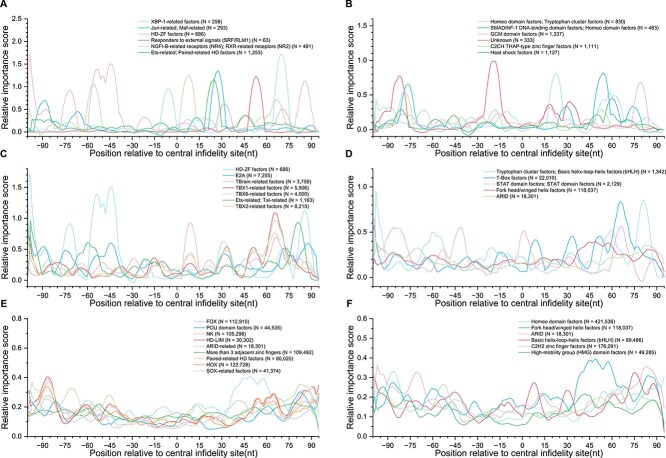
Analysis of the contribution of position-specific transcription factor families and classes to transcriptional infidelity sites. In all legends, the final ``N'' represents the total number of binding sites for the transcription factors within each family or class. (A and B) respectively show the top six categories within transcription factor families and classes that have the highest relative importance scores at specific individual sites. (C and D) respectively display the top seven families and top five classes based on the cumulative relative importance scores across all sites. (E and F) respectively illustrate the families and classes that exhibit positive importance scores across the entire distribution of sites.

We conducted a further classification analysis of transcription factors based on their families and classes using three analytical approaches: (i) Top individual sites: We identified the top six families and classes with the highest relative importance scores at specific individual sites (Fig. 3A and B). (ii) Cumulative importance scores: We identified the top seven families and top five classes based on the cumulative relative importance scores across all sites (Fig. 3C and D). (iii) Positive importance across all sites: We displayed families and classes that exhibit positive importance scores across the entire site distribution (Fig. 3E and F). To reduce noise in the data and facilitate the observation of distribution trends, we applied weighted smoothing with a window size of 10 during visualization. In Fig. 3A, despite the limited number of binding sites for transcription factors within these families, their binding motifs exhibited higher importance scores in specific relative regions of the infidelity sites. For instance, the HD-ZF factors family displayed higher contribution scores within the $-40$ nt to $-60$ nt range around the infidelity sites. In Fig. 3C, we observed that the positional contribution distributions for the HD-ZF factors and E2A families were quite similar, as were those for the TBrain-related factors, TBX6-related factors, TBX1-related factors, and TBX2-related factors families. Notably, the HD-ZF factors family ranked highly in both the first and second analytical approaches. In Fig. 3E, we observed transcription factor families that consistently contributed positively across the distribution of infidelity sites, including the NK, Paired-related HD factors, HOX, and HD-LIM families, which exhibited highly similar patterns across the entire distribution. Figure 3B, D, and F reveal similar distribution patterns when transcription factors were classified by class rather than by family. However, it is important to consider the value of *N* (as indicated in the legend, representing the total number of binding sites) when interpreting these distributions, as the *N* value can influence the scaling of the distribution. Detailed importance scores and frequency distributions for transcription factor families and classes are recorded in Supplementary Data 3.

Finally, we analyzed the core consensus motifs for the transcription factor families mentioned above. We found that the core consensus motif for the HD-ZF factors and E2A families was CACCTG, while the core consensus motif for the TBrain-related factors, TBX6-related factors, TBX1-related factors, and TBX2-related factors families was AGGTGTGA. The core consensus motif for the NK, Paired-related HD factors, HOX, and HD-LIM families was [C/T]AATTA. Additional information on the contributions of transcription factor families and classes can be found in Supplementary Data 2 and 3.

### Validation of the reliability of the interpretation scheme through basic sequence features

**Figure 4:**
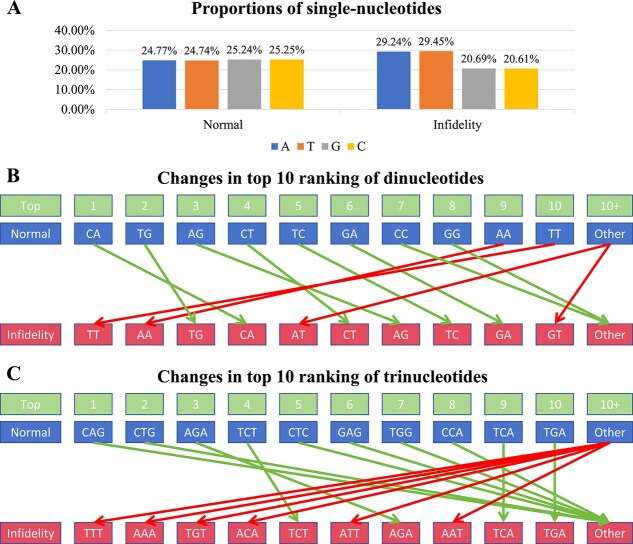
Analysis of basic sequence features for normal sequences and sequences with transcriptional infidelity used in training and interpretation analyses. (A) Proportions of single-nucleotides in normal and transcriptional infidelity sequences. (B and C) Changes in the top 10 ranking of dinucleotides and trinucleotides by frequency proportion, where green arrows indicate a decrease or no change in ranking from normal to transcriptional infidelity sequences, and red arrows indicate an increase in ranking, which may suggest a close association between these dinucleotides or trinucleotides and the occurrence of transcriptional infidelity.

To verify the reliability of the proposed interpretation scheme, we further analyzed the basic features of the sequences used for training and interpretation, including those containing regions of transcriptional infidelity and regions of normal transcription. These features cover the proportions of single-nucleotides, as well as the ranking changes in the proportions of dinucleotides and trinucleotides, as shown in Fig. 4. In Fig. 4A, we observed that in sequences with normal transcription, the proportions of the four single-nucleotides were relatively uniform, with almost no significant differences. However, in sequences with transcriptional infidelity, the AT content was significantly higher than the GC content, with a difference of $\sim $9 percentage points. This difference may suggest a correlation between the occurrence of transcriptional infidelity and the nucleotide composition of the sequence. In Fig. 4B and C, we further examined the ranking changes in the proportions of dinucleotides and trinucleotides between sequences with transcriptional infidelity and those with normal transcription. We noticed that the dinucleotides and trinucleotides whose rankings significantly increased in transcriptional infidelity sequences might be closely related to the occurrence of transcriptional infidelity.

Our deep learning framework and its interpretation scheme offer a deeper perspective for understanding the potential causes of transcriptional infidelity, and the interpretation results align closely with the findings from the basic sequence feature analysis. For example, the importance of dinucleotides TT, AA, AT, GT, and trinucleotides TGT, ATT, and AAT corresponds with the core recognition motifs of some key transcription factor families, such as AGGTGTGA (associated with the TBrain-related factors, TBX6-related factors, TBX1-related factors, and TBX2-related factors families) and [C/T]AATTA (associated with the NK, Paired-related HD factors, HOX, and HD-LIM families). Moreover, the interpretation results also revealed scenarios that contrasted with the trends observed in the basic sequence features. For instance, although the proportions of dinucleotides CA, TG, CT, CC, and the trinucleotide CTG decreased in transcriptional infidelity sequences, the interpretation results indicated that the motif CACCTG (a core motif for the HD-ZF factors and E2A families) played a significant role in transcriptional infidelity. Overall, the changes in basic sequence features further validate the reliability of the interpretation results. Additionally, these interpretative analyses can uncover specific phenomena that are challenging to capture through basic sequence feature analysis, providing a more comprehensive perspective for understanding the mechanisms underlying transcriptional infidelity.

## Discussion

In this study, we proposed a deep learning framework, TTIRI, designed to predict transcriptional infidelity regions within the allopolyploid line by goldfish and common carp hybrids. To the best of our knowledge, TTIRI is the first computational model to utilize genomic sequence information to locate regions of transcriptional infidelity. To accurately identify these regions, we employed a two-layer prediction strategy: an initial prediction at the sequence-level followed by a refined prediction for subregions. However, during the subregion prediction process, the challenge of data class imbalance could not be adequately addressed by common techniques of equal downsampling. Therefore, we introduced a weighted loss function to mitigate the negative impact of class imbalance on model performance. Through experimental validation, we identified the optimal balance between sequence length and subregion design, demonstrating that TTIRI can accurately identify transcriptional infidelity regions at both the sequence and subregion levels. To gain a deeper understanding of how the model captures transcriptional infidelity regions based on sequence information, we designed a systematic quantitative analysis scheme. Initially, we perturbed the position-specific hexamers at transcriptional infidelity sites and quantitatively analyzed their contributions to the predictions. However, due to the existence of as many as 4096 types of hexamers, directly analyzing the contribution of all hexamers proved to be both complex and inefficient. To enhance the quality and efficiency of our analysis, we introduced transcription factor recognition motifs, utilizing motif screening to narrow down the scope of analysis. We then further classified and analyzed the predictive contributions according to transcription factor families and classes. Notably, the interpretive analysis revealed that TTIRI successfully captured key transcription factor recognition motifs, such as the core motif CACCTG of the HD-ZF factors and the E2A families, along with their position-specific contribution characteristics. We further validated the reliability and insights of the interpretation results by examining the consistency between changes in basic sequence features and the interpretation outcomes. These findings indicate that TTIRI not only effectively predicts transcriptional infidelity regions in the allopolyploid line by goldfish and common carp hybrids, but its predictive results and interpretive analysis also provide new perspectives and understanding for a deeper exploration of the transcriptional infidelity phenomenon.

Despite the satisfactory performance of TTIRI, there is still room for further optimization. Currently, TTIRI uses only DNA context as input, which might limit the model’s ability to accurately characterize species genomes and their differentiated cellular states in the absence of genomic annotations, such as genomic regions, structures, and epigenetic information. For instance, studies have shown that integrating DNA structural information—such as minor groove width, propeller twist at base-pair resolution, roll, and helix twist—can significantly enhance model performance in DNA double-strand break prediction and reveal the critical role of DNA structure in lesion recognition [30]. Similarly, in CRISPR-induced double-strand break prediction, the inclusion of epigenetic features like CTCF, Dnase, H3K4me3, and RRBS as additional features has markedly improved predictive power, with ablation experiments further highlighting the importance of these chromatin status markers in the CRISPR-induced cleavage process [31, 32]. Moreover, TTIRI still has certain limitations regarding the granularity of subregion prediction, potentially stemming from the constraints of the model design and the influence of data class imbalance in subregions. Future versions of TTIRI will attempt to integrate more genomic information and refine the model architecture to achieve more precise predictions, ideally reaching single-nucleotide resolution, thereby enabling a deeper understanding of the regulatory mechanisms underlying transcriptional infidelity. Although there is still room for improvement in the current study, our findings strongly support the understanding of transcriptional infidelity patterns within the allopolyploid line by goldfish and common carp hybrids, showcasing the potential application value of this tool in related research. Future work will focus on enhancing TTIRI's predictive accuracy and interpretative capabilities, laying the foundation for an in-depth exploration of the molecular mechanisms underlying transcriptional infidelity phenomena.

## Conclusion

In this study, we present the first application of deep learning at nucleotide resolution to uncover patterns of transcriptional infidelity in the allopolyploid line by goldfish and common carp hybrids. We developed a two-layer computational framework (TTIRI model) that accurately identifies regions of transcriptional infidelity and devised an accompanying interpretability scheme to systematically quantify the positional contributions of hexamer motifs, transcription factor families, and classes to model predictions. Our findings provide novel insights into the pattern features and molecular mechanisms of transcriptional infidelity at nucleotide resolution, and offer valuable leads for dissecting the deeper factors driving this phenomenon. Moreover, this work contributes important understanding toward the molecular basis underlying the rarity, reduced viability, and limited reproduction of polyploidized vertebrates, thereby establishing a robust theoretical foundation for future research on vertebrate polyploidization.

**Key Points**

- First application of deep learning at nucleotide resolution to uncover pattern features of transcriptional infidelity.
- Development and implementation of a two-layer computational framework (TTIRI model) that accurately identifies regions of transcriptional infidelity.
- Establishment of an interpretability scheme that systematically quantifies the contributions of position-specific hexamers, transcription factor families, and classes to model predictions, revealing their associations with transcriptional infidelity.
- Provision of novel insights into the pattern features and molecular mechanisms of transcriptional infidelity at nucleotide resolution.
- Offering valuable leads and references for understanding the drivers of transcriptional infidelity and for future research on vertebrate polyploidization.

## Supplementary Material

Supplementary_Data_1_(Comparison_of_model_performance_under_different_settings)_bbaf260

Supplementary_Data_2_(Importance_scores_and_frequency_distribution_of_motifs)_bbaf260

Supplementary_Data_3_Importance_scores_and_frequency_bbaf260

Supplementary_File_bbaf260

## Data Availability

To facilitate replication of this study and further advancement in related research, we have made the data and code used for the models and interpretation methods developed in this work publicly available at https://github.com/KzJing/TTIRI.
