# Supplementary material for: Deep learning reveals determinants of transcriptional infidelity at nucleotide resolution in the allopolyploid line by goldfish and common carp hybrids
Source: Brief Bioinform. 2025 Jun 5;26(3):bbaf260. doi: 10.1093/bib/bbaf260 (PMC12140016; doi:10.1093/bib/bbaf260)
Supplement: Supplementary_File_bbaf260 [file supplementary_file_bbaf260.docx]

**Nomenclature (List of Abbreviations)**

**DNA**: deoxyribonucleic acid

**RNA**: ribonucleic acid

**RDDs**: RNA-DNA sequence differences

**TTIRI**: a two-layer transcriptional infidelity region identifier

**BERT**: Bidirectional Encoder Representations from Transformers

**NLP**: natural language processing

**BPE**: Byte-Pair Encoding

**CLS**: classification token

**SEP**: separator token

**ALiBi**: the attention with linear biases

**AUROC**: the area under the receiver operating characteristic curve

**AUPRC**: the area under the precision-recall curve

**PFM**: position frequency matrix

**PWM**: position weight matrix

**CNN**: Convolutional Neural Network

**LSTM**: Long Short-Term Memory

**GRU**: Gated Recurrent Unit

**Sequence-Specific Analysis Example**

Using the following 201 nt DNA sequence as an example, we applied the three analytical approaches described in the main text:

CAAGCAACAT TCCTTCAGGT ACAGAAGTTA TTTGTGCCAT GTACATCATC TAAACACTTT TTCGTCTTGG TTATCTCAGC AAACATTCTG TAAATTATTA G CAGGTTTGTC TGGGACAGAT ACAGAGAGTA TTGGGTTTTC TCAGGTATTT GCTAAAACCA GATGATTAAT TCTGTCCATT GGCCTTATTA TCACAAAACA

(Spaces inserted every 10 nt for readability; the “G” highlighted in yellow denotes the experimentally validated transcriptional infidelity site.)

1.**Top individual sites**

We identified the five transcription factor families and classes with the highest relative importance scores at individual nucleotide positions (Fig. S1A,B).

2.**Cumulative importance scores**

We computed the sum of relative importance scores across all positions, and thereby identified the top seven families and top five classes by cumulative score (Fig. S1C,D).

3.**Positive importance across all sites**

We display the top seven transcription factor families and top five classes whose importance scores remain positive over the greatest number of nucleotide positions (Fig. S1E,F). In this sequence-specific example, no family or class achieved positive importance at every position; accordingly, we show those with the maximal positive-coverage span.

All profiles were smoothed by applying a weighted moving average with a window size of 10 nt.

Figure S1 reveals four discrete regions of elevated importance in this sequence-specific example: –55 to –40 nt, –15 to 0 nt, +55 to +70 nt, and +80 to +90 nt. These intervals are highlighted in red in the DNA sequence above and are all notably AT-rich, consistent with the enrichment patterns shown in Figure 4A of the main text. However, because the number of transcription factor binding sites (N) in this individual sample is low, the observed associations between TF features and transcriptional infidelity may lack sufficient representativeness and thus warrant further experimental validation.





**Fig. S1.** Example of sequence-specific, position-specific contribution distributions of transcription factor families and classes to transcriptional infidelity sites. In all legends, the trailing “N” denotes the total number of binding sites for that TF family or class. **A, B.** The five families (A) and five classes (B) with the highest relative importance scores at individual positions. **C, D.** The seven families (C) and five classes (D) with the highest cumulative importance scores summed over all positions. **E, F.** The top seven families (E) and top five classes (F) whose importance scores remain positive across the greatest number of positions.

**Two-layer architecture design advantage**

To validate the advantage of the proposed two-layer architecture for identifying transcriptional infidelity regions, we conducted a comparative evaluation between two models (see Fig. S2): a single-layer model (referred to as TTIRI-S), which directly integrates DNABERT2 with the subregion identification module (including merge tokens block and subregion-level classifier), and the two-layer model (TTIRI). To ensure a fair comparison and mitigate the impact of class imbalance, both models were evaluated using the area under the precision-recall curve (AUPRC) metric. It is important to note that TTIRI-S was selected based on the maximum subregion-level AUPRC, whereas TTIRI was selected based on the sequence-level AUROC; thus, the comparison based on subregion-level AUPRC is relatively conservative for TTIRI.

As shown in the results, across various subregion sizes (i.e., different values of M-tokens), the two-layer architecture consistently outperforms the single-layer model, with an average improvement of approximately 6 percentage points. The advantage of the two-layer design primarily lies in its ability to filter out a large number of entirely negative samples (i.e., sequences without any transcriptional infidelity) at the sequence level, thereby simplifying the subregion-level prediction task. As indicated in Table S1, adopting the two-layer architecture leads to a marked increase in the proportion of positive samples within subregions, which helps alleviate class imbalance and reduce bias. Furthermore, the sequence-level predictions alone already achieve strong performance, and the two-layer architecture effectively propagates this stability and predictive strength to the subregion level. This facilitates more stable model training and convergence, ultimately enhancing overall predictive performance.

It is worth noting that the computational complexity of both TTIRI-S and TTIRI is primarily determined by the underlying Transformer architecture, which exhibits a quadratic growth with respect to sequence length (O(n²)). As a result, the time consumption of the two models is comparable. Under our experimental configuration—using four Tesla V100 GPUs, a 36-core CPU, and 192 GB of memory—and training setup (40 epochs), each run takes approximately 20 minutes, including job scheduling time on the high-performance computing cluster. The actual runtime of the program is approximately 13 minutes.


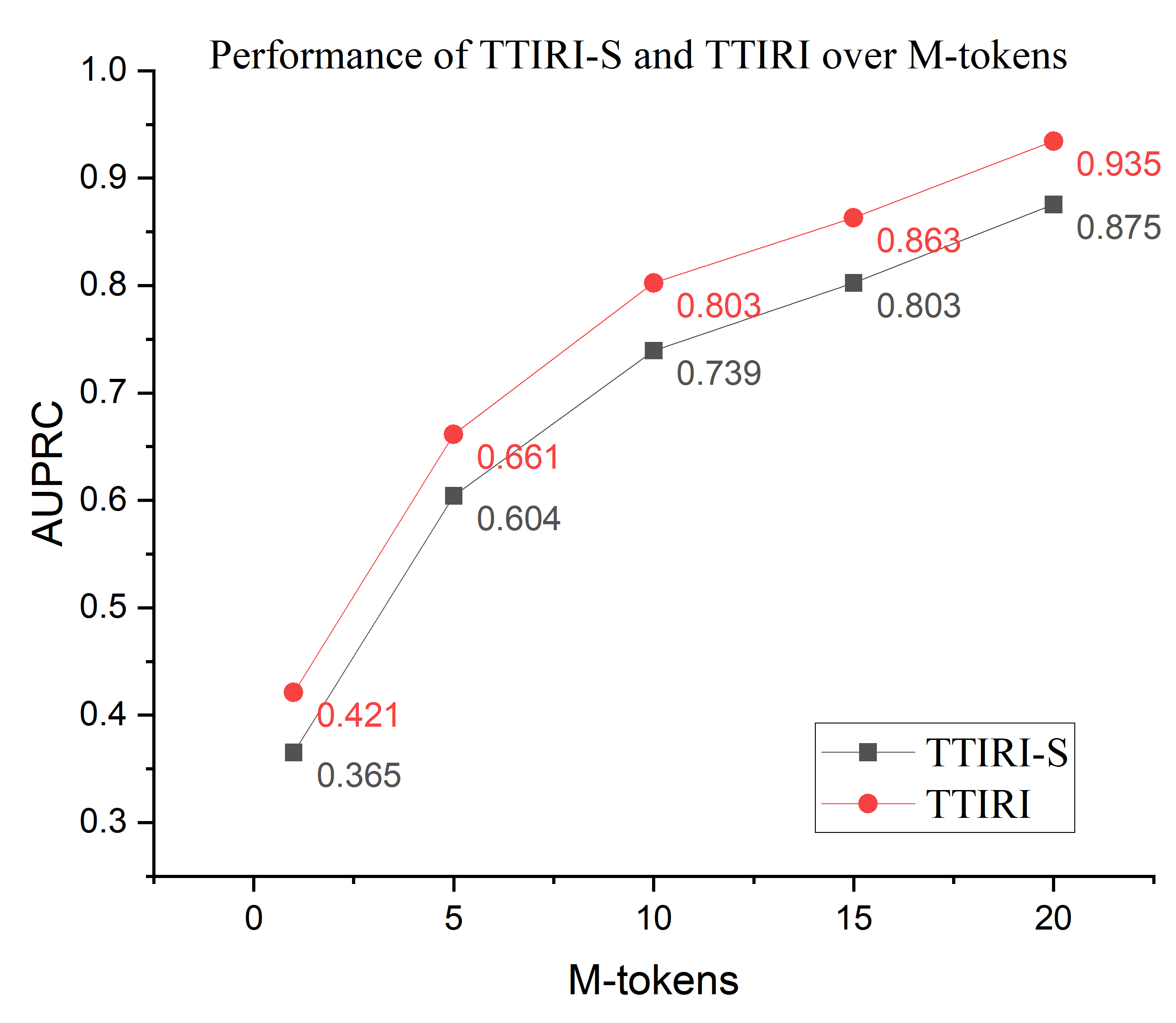


**Fig. S2.** Prediction performance comparison between TTIRI-S and TTIRI across different M-token values.

**Tab. S1.** Proportion of positive samples in subregions for TTIRI-S and TTIRI across different M-token values.

| **M-tokens** | **Proportion of positive samples in subregions** | |
| --- | --- | --- |
|  | **TTIRI-S** | **TTIRI** |
| 1(~5 nt) | 4.9% | 9.1% |
| 5(~25 nt) | 16.5% | 31.3% |
| 10(~50 nt) | 24.2% | 45.8% |
| 15(~75 nt) | 32.0% | 58.8% |
| 20(~100 nt) | 39.9% | 71.8% |
